# Supplementary figures and images for: Kinetics of Plasmodium midgut invasion in Anopheles mosquitoes
Source: PLoS Pathog. 2020 Sep 18;16(9):e1008739. doi: 10.1371/journal.ppat.1008739 (PMC7526910; doi:10.1371/journal.ppat.1008739)

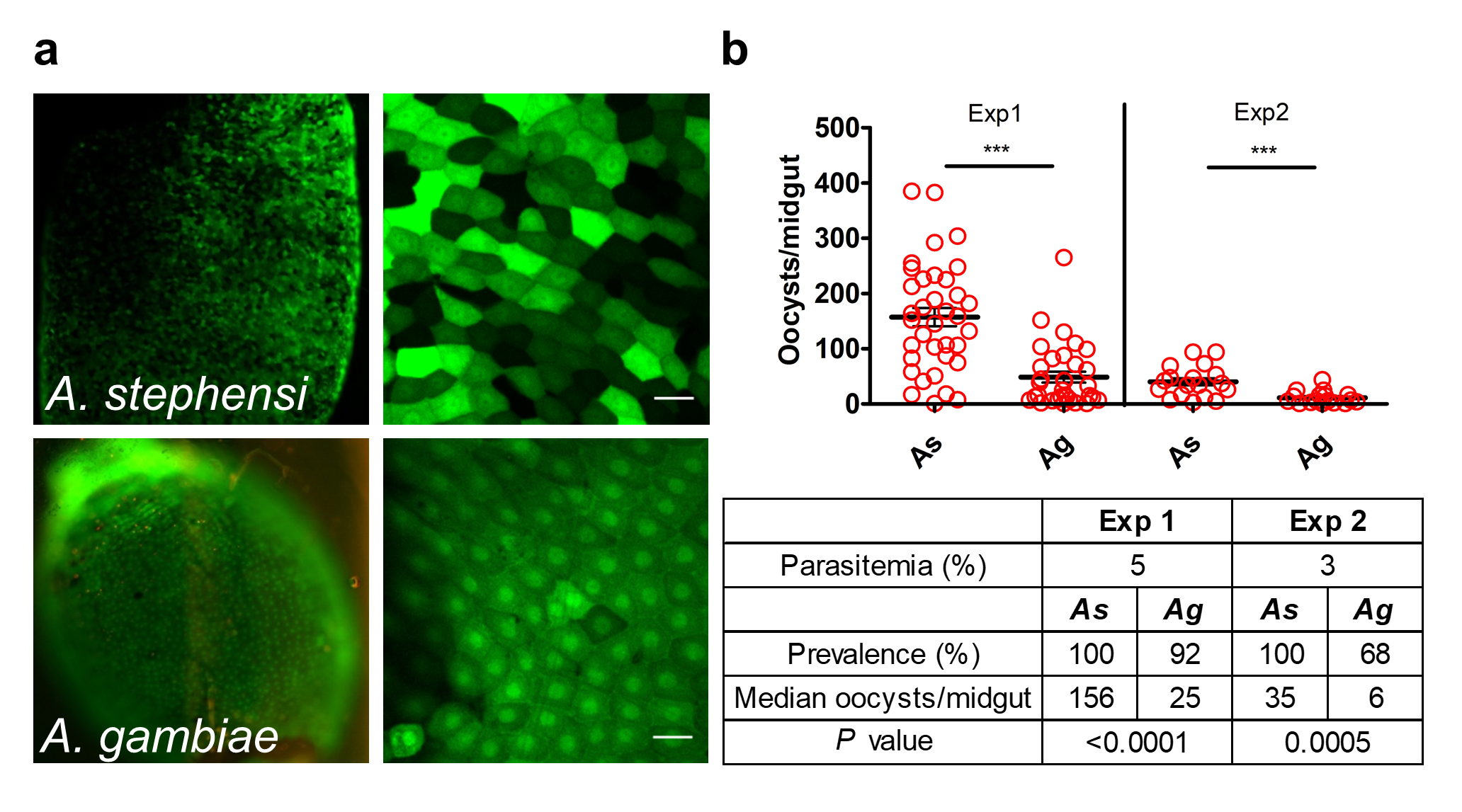

Supplement: S1 Fig — a. GFP fluorescence in the midgut cells of A. stephensi G12::GFP line (upper) and A. gambiae dmActin5c::dsx-eGFP line (lower) 24 h after blood feeding. Enlarged are representative 20-fold magnification images showing GFP fluorescence in enterocytes (scale bar—50 μm). b. P. berghei infection intensities in A. stephensi and A. gambiae. Oocysts were counted in dissected midguts 7 days post infection. The results of two independent experiments are shown. Prevalence indicates the percentage of infected mosquito midguts in each experiment. Horizontal lines depict median number of oocysts per midgut. Statistical differences between infections of As and Ag were evaluated by a nonparametric t test, *** indicate P < 0.0005. (TIF) [file ppat.1008739.s001.tif]

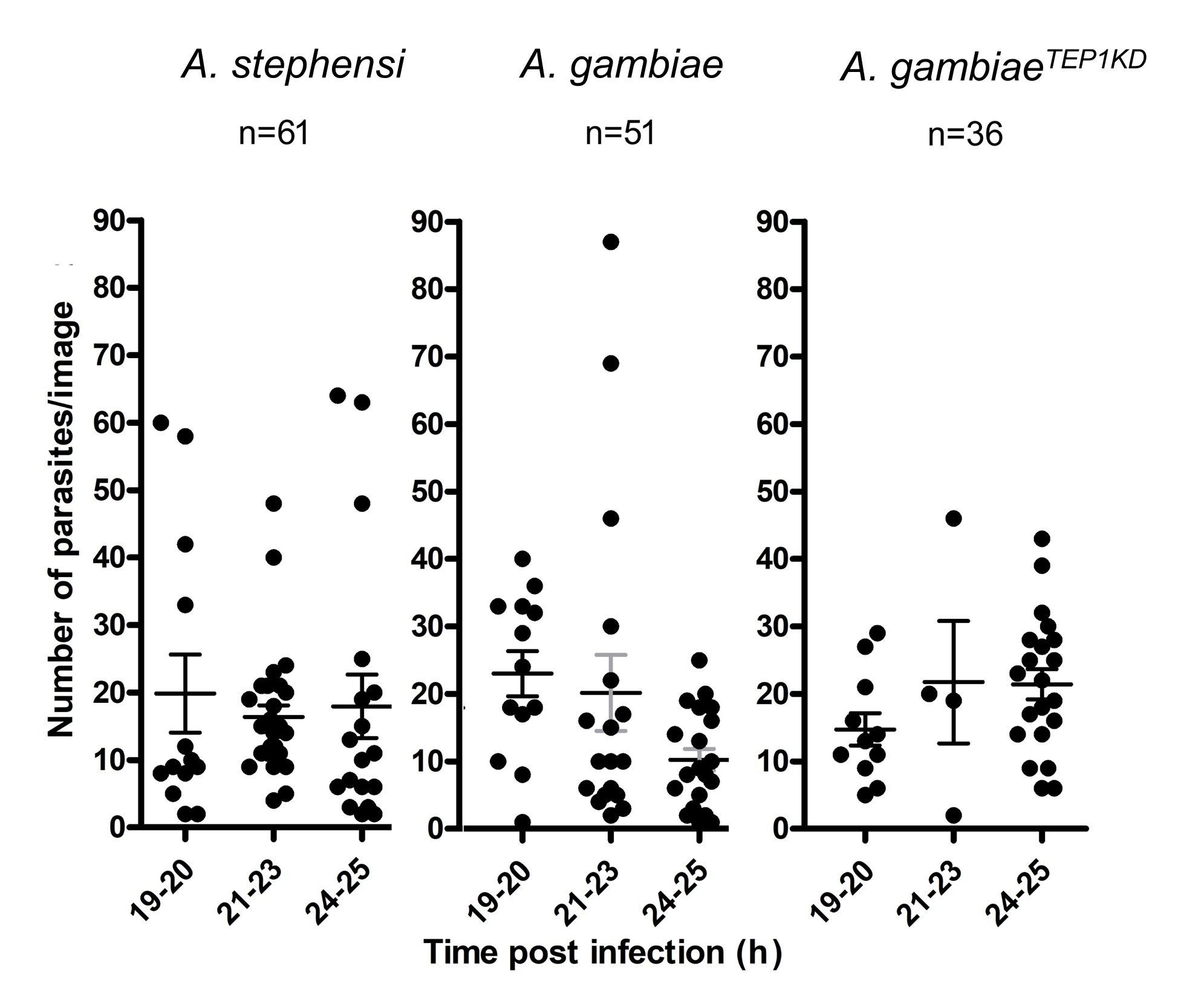

Supplement: S2 Fig — Each dot represents a single midgut. Similar numbers of parasites were analyzed in all mosquitoes at the indicated time points (h) after infection (hpi), where n is the number of analyzed midguts. (TIF) [file ppat.1008739.s002.tif]

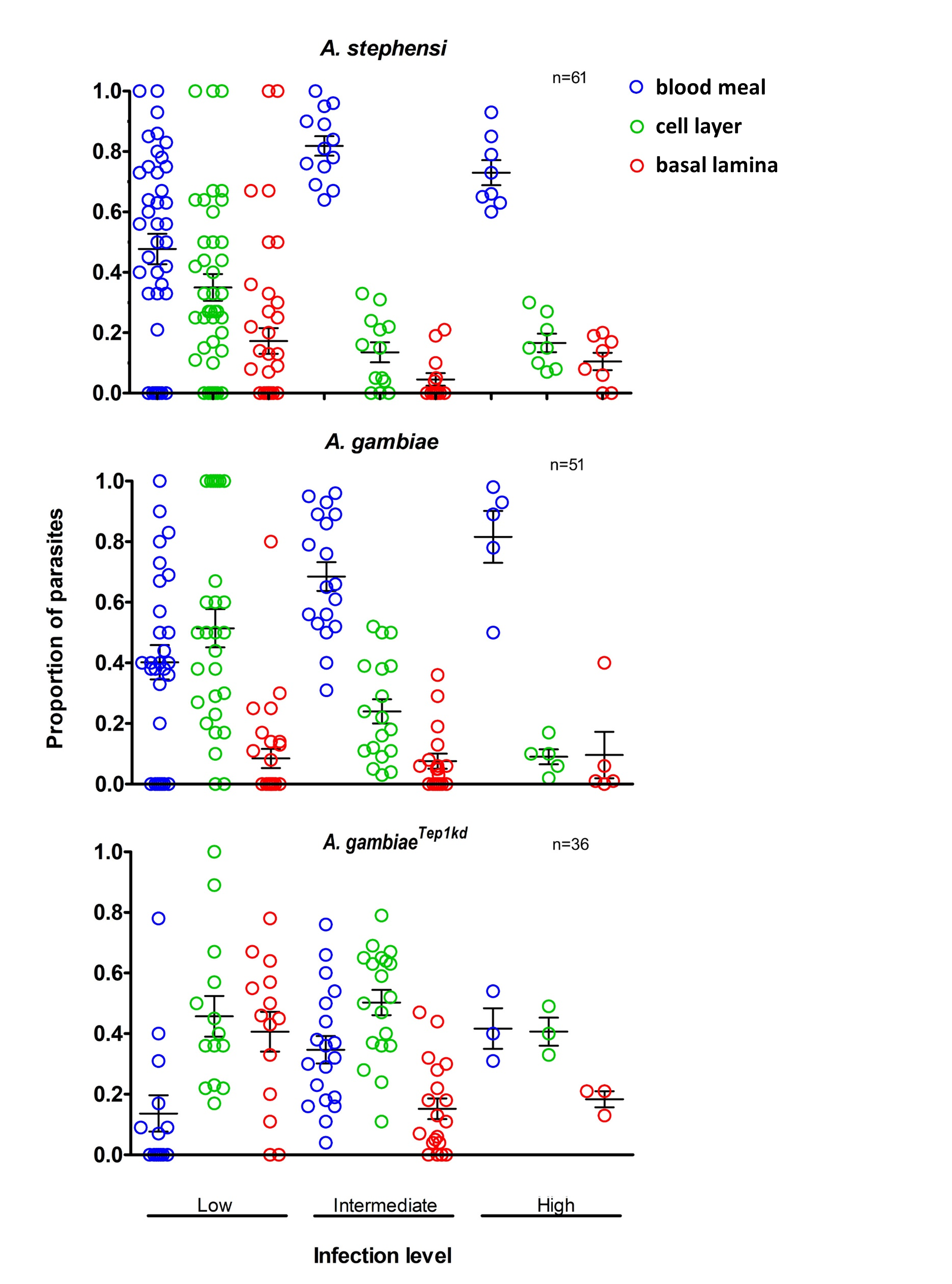

Supplement: S3 Fig — Localization of parasites in the blood meal (blue), cell layer (green) and basal lamina (red) in the midguts grouped by the infection level. Low infection (up to 15 parasites), intermediate (16–35 parasites) and high (more than 35 parasites per image) are compared. Each dot represents the proportion of parasites at a given position in a single midgut. n is the number of analyzed images. (TIF) [file ppat.1008739.s003.tif]

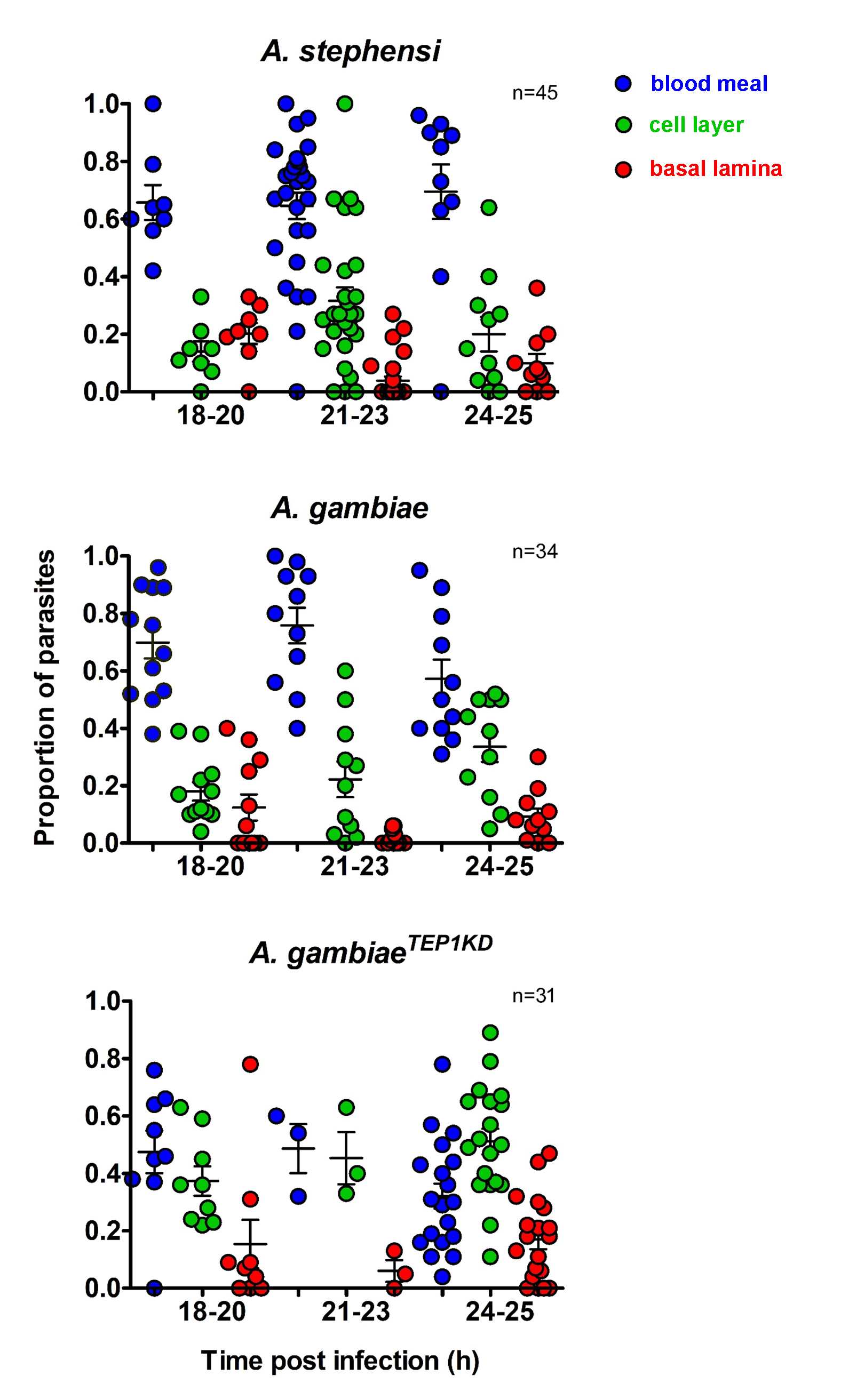

Supplement: S4 Fig — Proportion of parasites found in the blood meal (blue), cellar layer (green) and basal lamina (red) at the indicated time points (h) after infection (hpi). N is the number of analyzed images. All analyzed images contained at least ten parasites. (TIF) [file ppat.1008739.s004.tif]

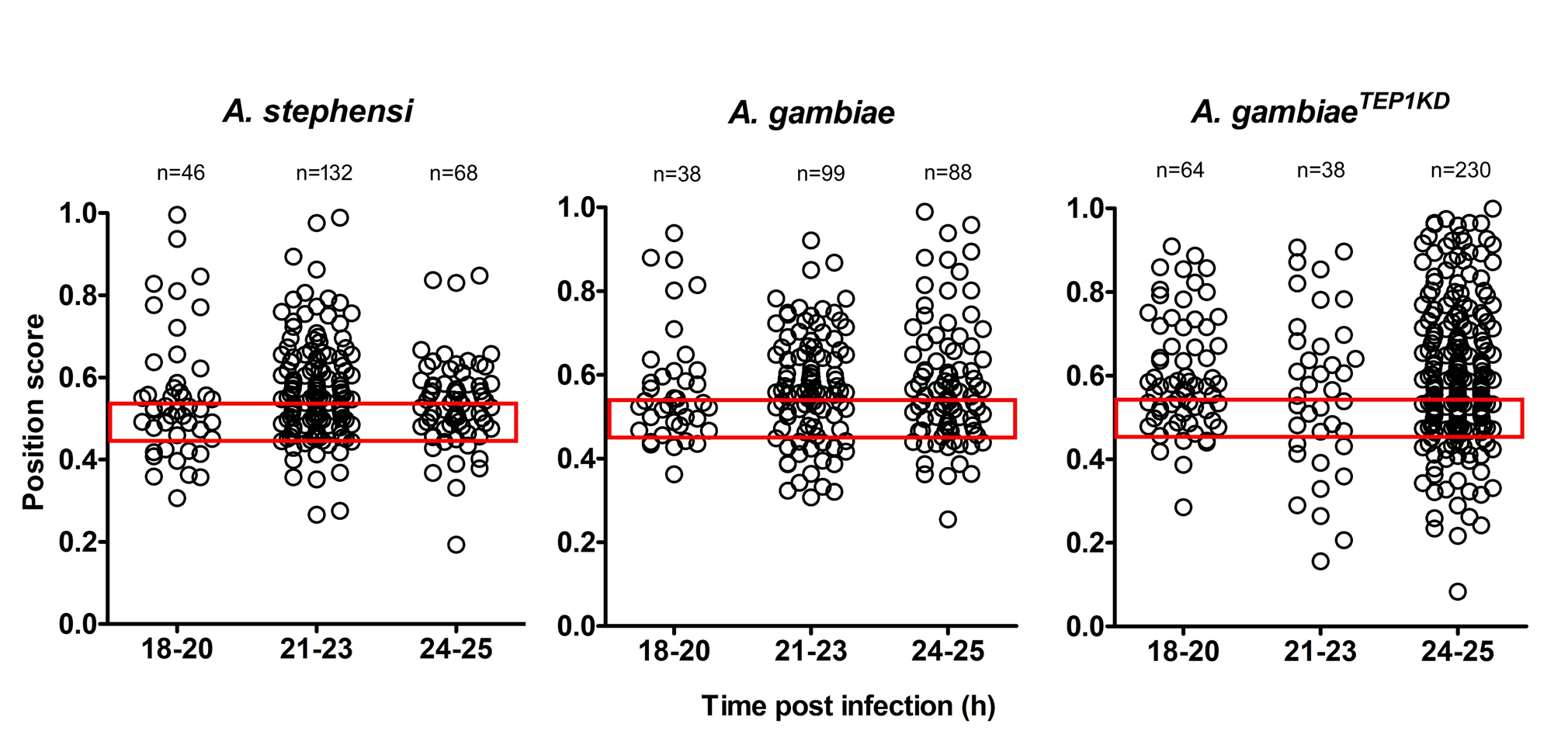

Supplement: S5 Fig — Scatter plots depict the score for each parasite at indicated times after infection. Parasites are considered extracellular when the score s < 0.45, intercellular for the score 0.45 < s < 0.55 (red box) and intracellular if the score s > 0.55. n is the number of parasites analyzed at each time interval. (TIF) [file ppat.1008739.s005.tif]

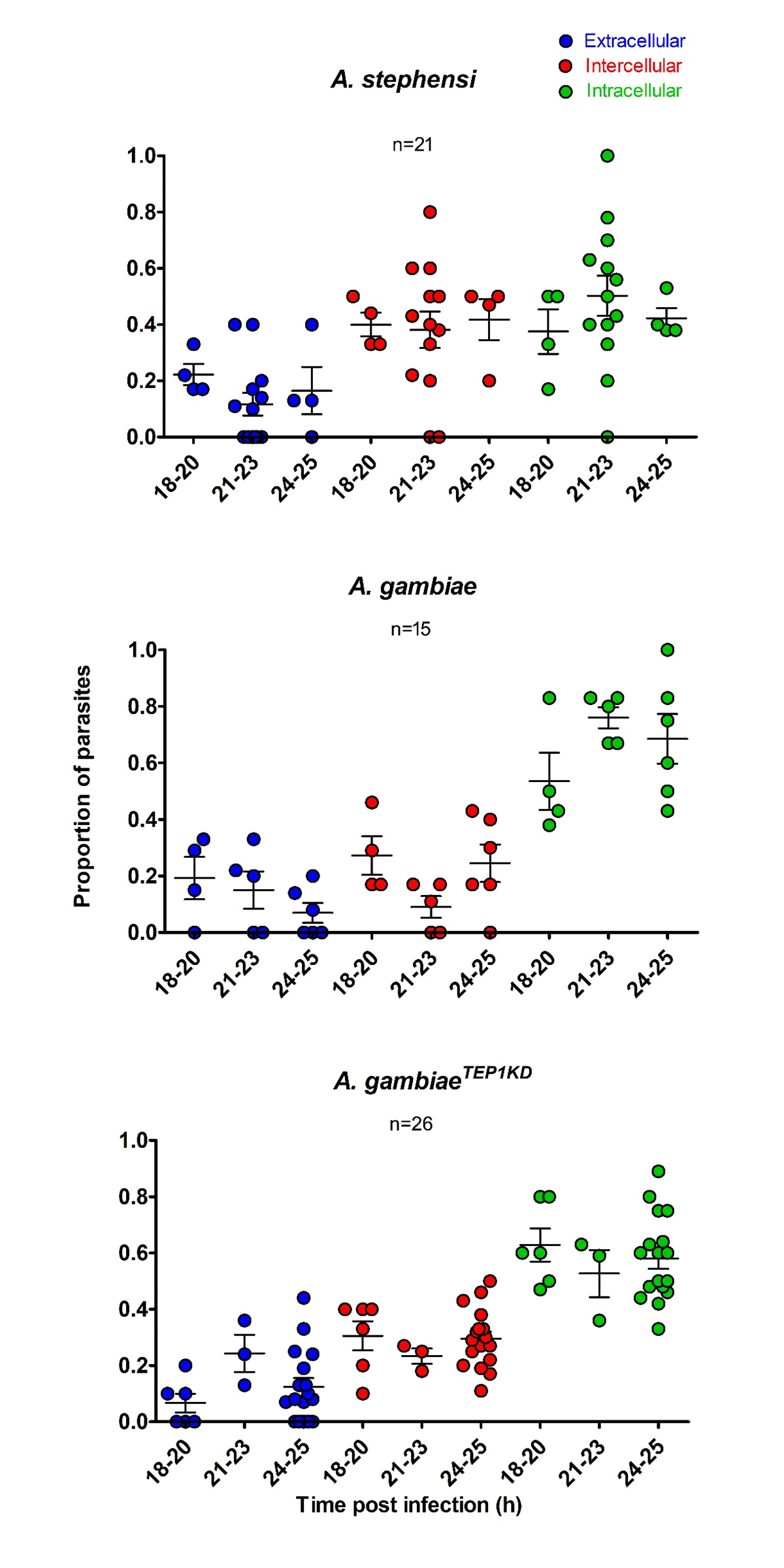

Supplement: S6 Fig — Scatter plots depict the proportion of parasites at each position within the cell layer: extracellular (blue), intercellular (red) and intracellular (green) at different time intervals after infection. Each dot represents a single image which contained at least 6 parasites in the cellular layer. n is the number of analyzed images. (TIF) [file ppat.1008739.s006.tif]

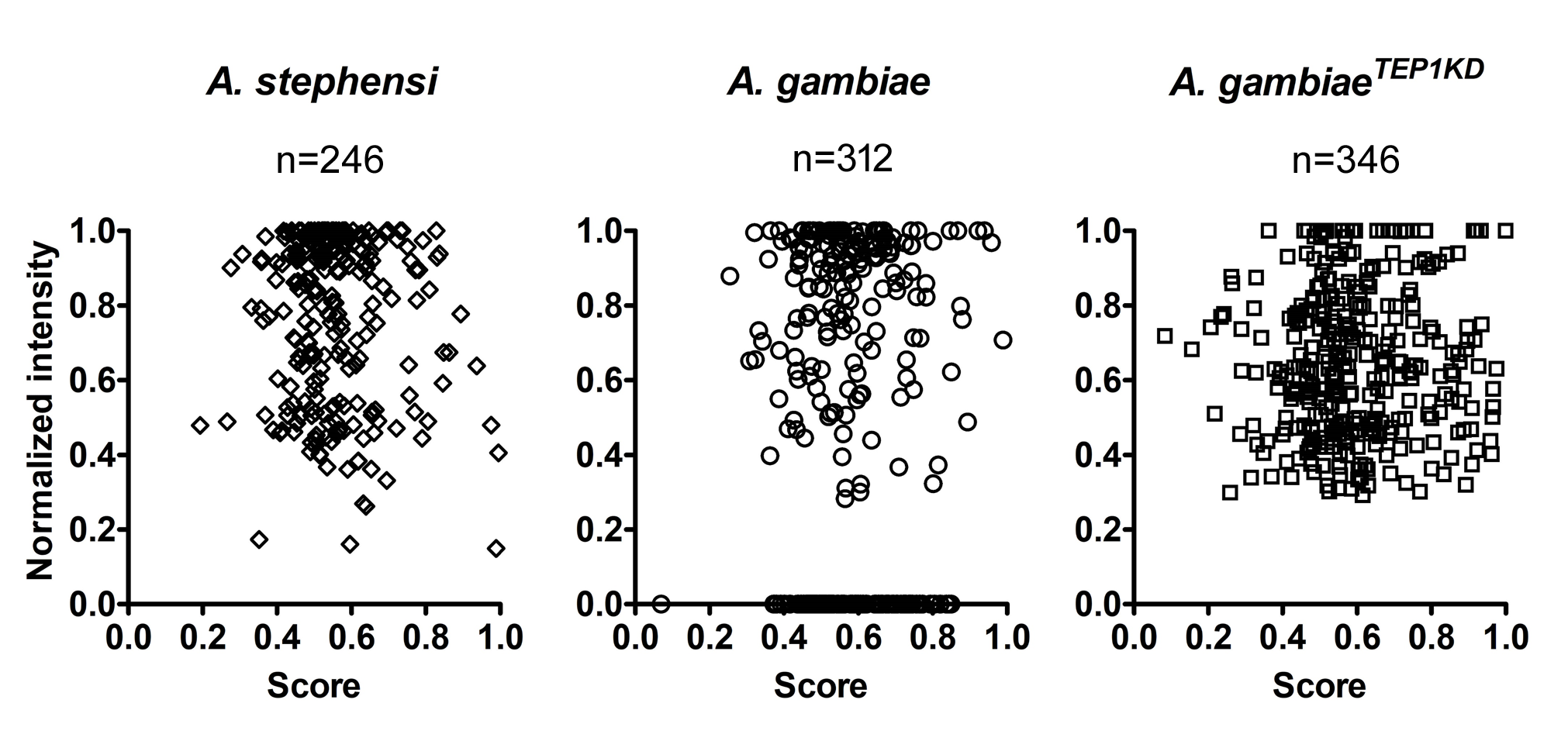

Supplement: S7 Fig — Each dot represents a parasite. n is the number of parasites depicted. No correlation was found between the position of the parasite and the level of fluorescence intensity when intensity is greater than zero. (TIF) [file ppat.1008739.s007.tif]

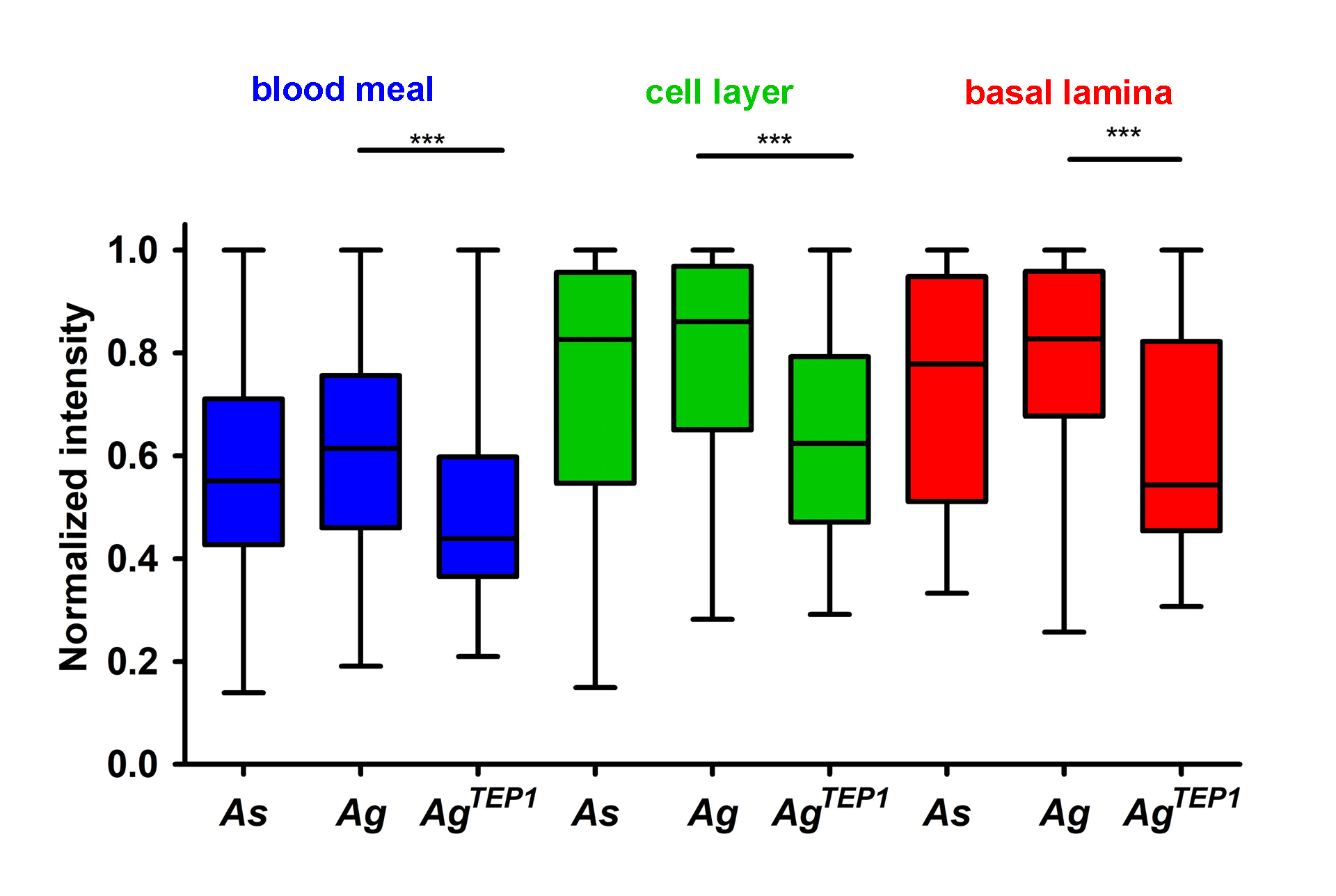

Supplement: S8 Fig — Bar graphs depict the distribution of parasite fluorescence intensity at different positions: blood meal (blue), cell layer (green) and basal lamina (red). Parasites from all time points were pooled to calculate the average normalized intensity. Parasite intensity is normalized for each image, intensity ranges between 0.0 and 1.0, where 1.0 is the maximum intensity observed. Statistical significance of differences within each group was tested by one-way ANOVA, and differences supported by P < 0.0001 were considered significant. (TIF) [file ppat.1008739.s008.tif]

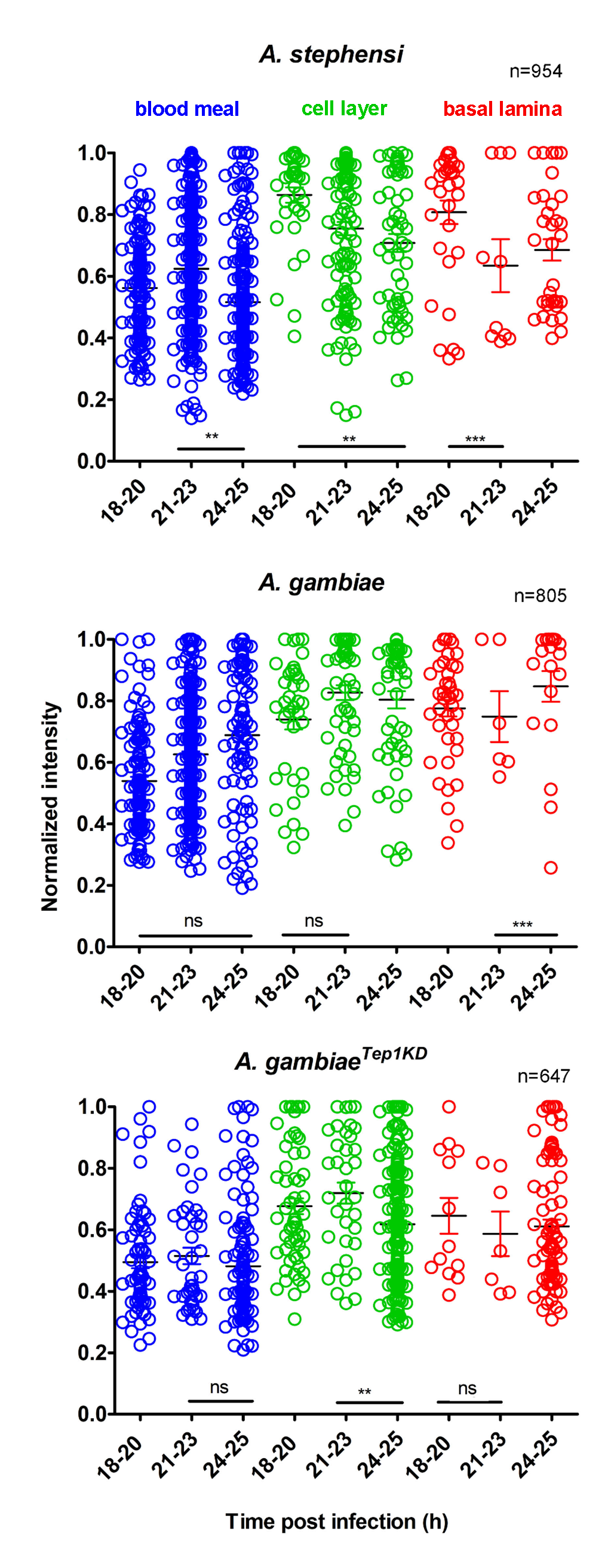

Supplement: S9 Fig — Parasite fluorescence intensity in A. stephensi (As), A. gambiae (Ag), and A. gambiae depleted for TEP1 (AgTEP1KD) at different times after infection and at different positions: blood meal (blue), cell layer (green) and basal lamina (red). Each circle represents a parasite. n is the number of analyzed parasites. Statistical analysis was performed by non-parametrical Mann Whitney test. Only images with more than 10 parasites were analyzed. (TIF) [file ppat.1008739.s009.tif]

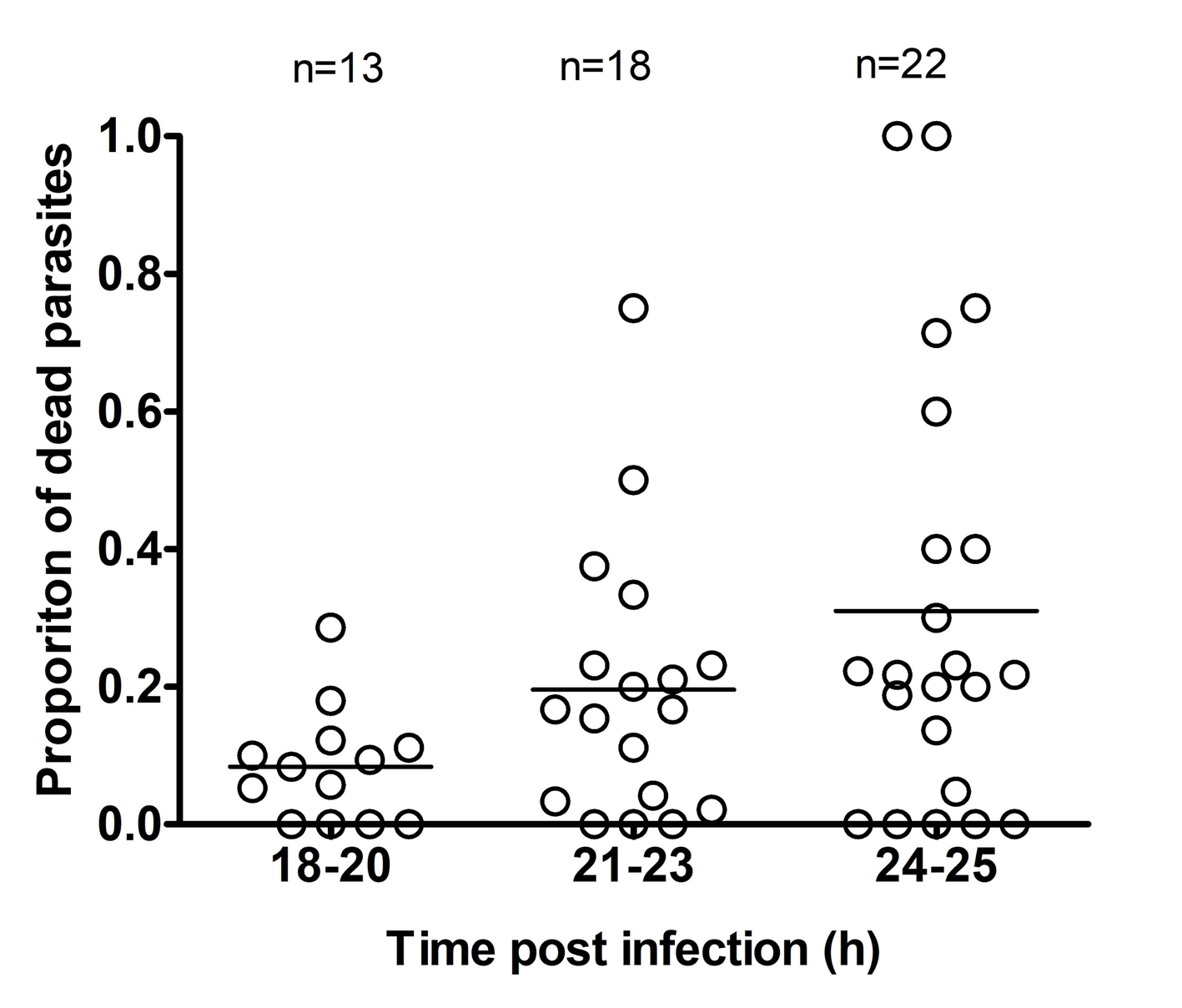

Supplement: S10 Fig — The proportion of parasites that are considered dead in each image at indicated time intervals after infection. Each dot represents one image, n is the number of analyzed images. All midguts were used for analysis. (TIF) [file ppat.1008739.s010.tif]

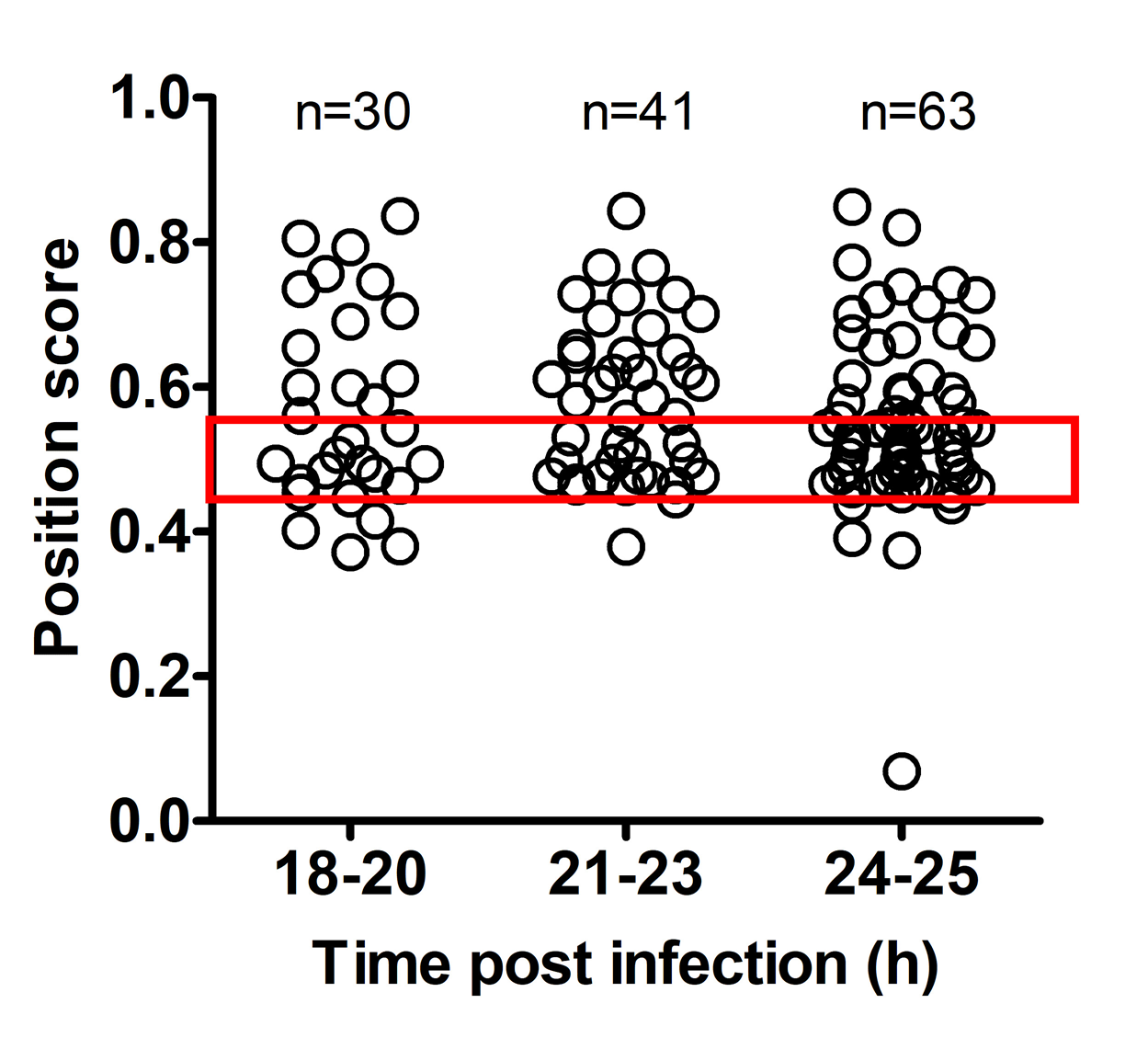

Supplement: S11 Fig — Scatter plots depict the score for each parasite at indicated times after infection. Parasites are considered extracellular when the score s < 0.45, intercellular for the score 0.45 < s < 0.55 (red box) and intracellular if the score s > 0.55. n is the number of parasites analyzed at each time interval. (TIF) [file ppat.1008739.s011.tif]

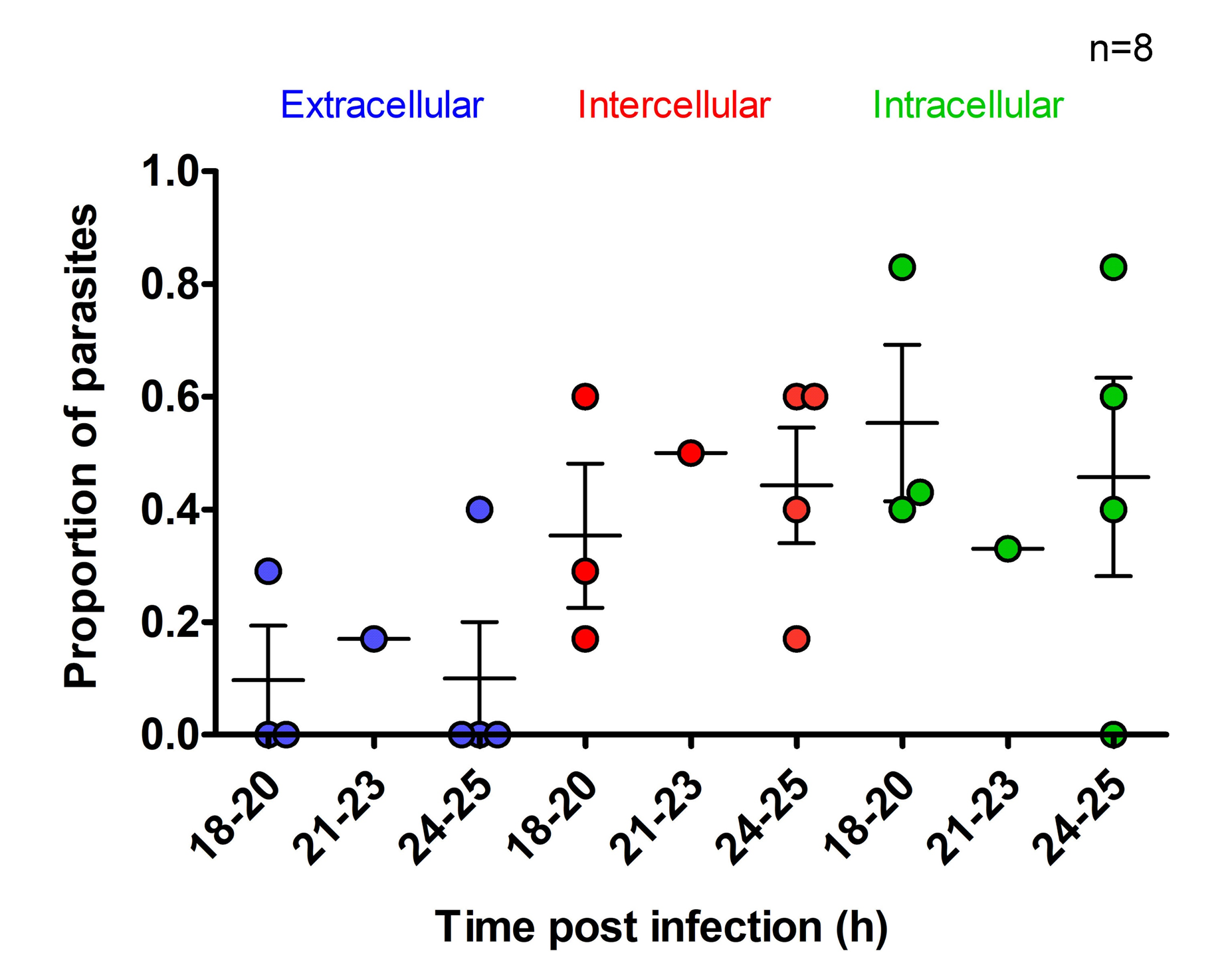

Supplement: S12 Fig — Scatter plots depict the proportion of parasites at each position within the cell layer: extracellular (blue), intercellular (red) and intracellular (green) at different time intervals after infection. Each dot represents a single image, n is the number of analyzed images. (TIF) [file ppat.1008739.s012.tif]
