# Supplementary material for: Kinetics of Plasmodium midgut invasion in Anopheles mosquitoes
Source: PLoS Pathog. 2020 Sep 18;16(9):e1008739. doi: 10.1371/journal.ppat.1008739 (PMC7526910; doi:10.1371/journal.ppat.1008739)
Supplement: S1 Table — (PDF) [file ppat.1008739.s013.pdf]

**Table S1.** Time-lapse records of ookinete invasion of *As* midguts in the presence or absence of dextran, the marker of cell membrane integrity. guid - the unique record number in the database, TPI - hours post infection, duration - the total time of the time-lapse measurement.

| guid | dextran | TPI (h) | duration(min) | feature | link |
|---|---|---|---|---|---|
| 2114 | no | 19.0 | 22 | **Z-project** | https://youtu.be/1_KEdIZWcqE |
| 2115 | no | 20.0 | 120 | **Z-project** | https://youtu.be/wkKHlYjaFwI |
| 1680 | no | 20.0 | 89 | **Z-project** | https://youtu.be/jfSG_acwGB0 |
| 1774 | no | 20.0 | 85 | **Z-project** | https://youtu.be/ROu7OrQ2w6I |
| | | | | **overview** | https://youtu.be/xVSNqvvbvDw |
| | | | | sideview | https://youtu.be/Up-RM_cicGo |
| | | | | sideview | https://youtu.be/w7TyNLBEeWA |
| | | | | sideview | https://youtu.be/a1HRxJlrzQU |
| | | | | zoom in | https://youtu.be/GDONJcZcCzA |
| 2107 | yes | 20.0 | 92 | **Z-project** | https://youtu.be/1vVwNaXOGYs |
| - | yes | 22.0 | 120 | **Z-project** | https://youtu.be/uRYpQGjh_vA |
| 2112 | no | 22.0 | 112 | **Z-project** | https://youtu.be/Hck5ksxpRvs |
| | | | | **overview** | https://youtu.be/SDpQ8KHQ2_M |
| | | | | zoom in | https://youtu.be/iaDC58Netlk |
| | | | | zoon in, rotate | https://youtu.be/DNb44lSTn8M |
| 1775 | no | 22.5 | 42 | **Z-project** | https://youtu.be/IsNrlOz1t-w |
| | | | | **overview** | https://youtu.be/C8mHzXEqIXo |
| | | | | zoom in | https://youtu.be/TJ8e3HhbxRk |
| | | | | zoom in | https://youtu.be/xsiwNR9zAPM |
| | | | | zoom in | https://youtu.be/1NBKAUeoBAo |
| 2119 | no | 22.5 | 95 | **Z-project** | https://youtu.be/ethg8Awj5FU |
| | | | | Overview + sideview | https://youtu.be/yGCnEb8UvJs |
| | | | | zoom in | https://youtu.be/FQy9jCkCalk |
| 1628 | no | 22.5 | 115 | **Z-project** | https://youtu.be/VpoFhhtUze0 |
| 1679 | no | 24.0 | 54 | **Z-project** | https://youtu.be/voohki6Qt0o |
| 2111 | | | | **Z-project** | https://youtu.be/52ZajjJXpsc |
| | | | | overview | https://youtu.be/Nl2Wk5zjnNw |
| | | | | sideview | https://youtu.be/bd-qGHj4KJ0 |
| 1777 | no | 24.0 | 58 | **Z-project** | https://youtu.be/NdMDozl9w5M |
| | | | | zoom in | https://youtu.be/UHNmziUt7iM |
| 1615 | no | 24.5 | 33 | **Z-project** | https://youtu.be/QlQrrtWgp9Q |
| | | | | sideview | https://youtu.be/r6Wcr7D10oo |
| | | | | overview | https://youtu.be/X1E31lS5gUA |
| 2113 | no | 24.5 | 49 | **Z-project** | https://youtu.be/DXH4xMxqCHc |
| - | no | 26.0 | 120 | **Z-project** | https://youtu.be/Z-zhEGavE34 |
| | | | | overview | https://youtu.be/oGMtzqZduJ8 |
| | | | | sideview | https://youtu.be/iO5OP0srrVw |
