# Supplementary material for: Kinetics of Plasmodium midgut invasion in Anopheles mosquitoes
Source: PLoS Pathog. 2020 Sep 18;16(9):e1008739. doi: 10.1371/journal.ppat.1008739 (PMC7526910; doi:10.1371/journal.ppat.1008739)
Supplement: S2 Table — (PDF) [file ppat.1008739.s014.pdf]

Table S2. Time-lapse records of ookinete invasion of *Ag* midguts in the presence of dextran, the marker of cell membrane integrity. Guid - the unique record number in the database, TPI - hours post infection, duration - the total time of the time-lapse measurement.

guid	dextran	TPI	duration (min)	feature	link
1796	Yes	19.0	50	**Z-project**	*https://youtu.be/RuxaIPgHywo*
1621	Yes	20.0	0	rotation of a single image	*https://youtu.be/ie-9RJ7t_pg*
2110	Yes	20.5	77	**overview**	*https://youtu.be/Zffuz1oevKk*
1622	Yes	21.5	48	**Z-project**	*https://youtu.be/4I5wZgSPOzA*
2109	Yes	22.0	31	**Z-project**	*https://youtu.be/m3VRyDDcxl4*
1624	Yes	22.5	20	**Z-project**	*https://youtu.be/bNN1Bu6-fOA*
2108	Yes	24.5	63	**Z-project**	*https://youtu.be/UlK_dlvEHts*
				diagonal view	*https://youtu.be/KRtBQAXlR3A*
				side view	*https://youtu.be/qnBLe2kpWGM*
