# Supplementary material for: Kinetics of Plasmodium midgut invasion in Anopheles mosquitoes
Source: PLoS Pathog. 2020 Sep 18;16(9):e1008739. doi: 10.1371/journal.ppat.1008739 (PMC7526910; doi:10.1371/journal.ppat.1008739)
Supplement: S3 Table — (PDF) [file ppat.1008739.s015.pdf]

Table S3. Kruskal-Wallis test of differences in ookinete localization between *A. gambiae* (*Ag*) and *A. gambiae* with silenced *TEP1* (*Ag*^{TEP1KD}) at the indicated time points post infection (hpi).

Ookinete distribution	Kruskal Wallis test		
	Ag	***Ag*^{TEP1KD}**	*P* value
**18-20 hpi**			
blood Meal	ns	ns	0.2186
cell Layer	< *Ag*^{TEP1KD}	> *Ag*	0.0418
basal Lamina	ns	ns	0.8842
**21-23 hpi**			
blood Meal	> *Ag*^{TEP1KD}	<*Ag*	0.064
cell Layer	< *Ag*^{TEP1KD}	> *Ag*	0.0826
basal Lamina	ns	ns	0.4561
**22-25 hpi**			
blood Meal	> *Ag*^{TEP1KD}	< *Ag*	0.0049
cell Layer	< *Ag*^{TEP1KD}	> *Ag*	0.0217
basal Lamina	ns	ns	0.5752
