# Supplementary material for: Kinetics of Plasmodium midgut invasion in Anopheles mosquitoes
Source: PLoS Pathog. 2020 Sep 18;16(9):e1008739. doi: 10.1371/journal.ppat.1008739 (PMC7526910; doi:10.1371/journal.ppat.1008739)
Supplement: S4 Table — (PDF) [file ppat.1008739.s016.pdf]

Table S4

. Kruskal-Wallis test of differences in parasite localization in *A. stephensi* (*As*), *A. gambiae* (*Ag*) and *A. gambiae* silenced for *TEP1* (Ag^{TEP1KD}) between the indicated time points after infection (hpi).

Ookinete position			Kruskal Wallis test	
	**18-20 hpi**	**21-23 hpi**	**24-25 hpi**	*P* value
As				
blood meal	ns	ns	ns	0.5852
cell layer	ns	ns	ns	0.1077
basal lamina	> 21-23 hpi	< 18-20 hpi<br>< 24-25 hpi	>21-23 hpi	0.0005
Ag				
blood meal	ns	>24-25 hpi	< 21-23 hpi	0.0369
cell layer	ns	< 24-25 hpi	> 21-23 hpi	0.0634
basal lamina	ns	< 24-25 hpi	> 21-23 hpi	0.0415
Ag^{TEP1KD}				
blood meal	> 24-25 hpi	ns	< 18-20 hpi	0.044
cell layer	< 24-25 hpi	ns	> 18-20 hpi	0.0499
basal lamina	ns	ns	ns	0.6019
