# Supplementary material for: Kinetics of Plasmodium midgut invasion in Anopheles mosquitoes
Source: PLoS Pathog. 2020 Sep 18;16(9):e1008739. doi: 10.1371/journal.ppat.1008739 (PMC7526910; doi:10.1371/journal.ppat.1008739)
Supplement: S5 Table — (PDF) [file ppat.1008739.s017.pdf]

Table S5. Kruskal-Wallis test of differences in parasite localization between *A. stephensi* (*As*), *A. gambiae* (*Ag*) and *A. gambiae* silenced for *TEP1* (Ag^{TEP1KD}) at the indicated time points after infection (hpi).

Ookinete position			Kruskal Wallis test	
	As	***Ag***	$\boldsymbol{Ag^{TEP1KD}}$	*P* value
**18-20 hpi**				
blood Meal	ns	ns	ns	0.3457
cell Layer	ns	ns	ns	0.1291
basal Lamina	ns	ns	ns	0.4012
**21-23 hpi**				
blood Meal	ns	$>Ag^{Tep1KD}$	$<Ag$	0.0833
cell Layer	ns	ns	ns	0.125
basal Lamina	ns	ns	ns	0.3879
**24-25 hpi**				
blood Meal	$> Ag^{TEP1KD}$	$> Ag^{TEP1KD}$	$< Ag, < As$	0.0033
cell Layer	$< Ag^{TEP1KD}$	$< Ag^{TEP1KD}$	$> Ag, > As$	0.0001
basal Lamina	ns	ns	ns	0.7941
