# Supplementary material for: Kinetics of Plasmodium midgut invasion in Anopheles mosquitoes
Source: PLoS Pathog. 2020 Sep 18;16(9):e1008739. doi: 10.1371/journal.ppat.1008739 (PMC7526910; doi:10.1371/journal.ppat.1008739)
Supplement: S6 Table — (PDF) [file ppat.1008739.s018.pdf]

Table S6. Kruskal-Wallis test of differences in parasite fluorescence intensities in *A. stephensi* (*As*), *A. gambiae* (*Ag*) and *A. gambiae* silenced for *TEP1* (*Ag*^{TEP1KD}) between the indicated time points after infection (hpi).

Ookinete fluorescence intensity				Kruskal Wallis test
	**18-20 hpi**	**21-23 hpi**	**24-25 hpi**	*P* value
As				
blood meal	>24-25 hpi<br><21-23 hpi	>18-20 hpi<br>>24-25 hpi	<18-20 hpi<br><21-23 hpi	
cell layer	>24-25 hpi	ns	<18-20 hpi	0.01
basal lamina	>24-25 hpi	ns	<18-20 hpi	0.037
Ag				
blood meal	<21-23 hpi<br>< 24-25 hpi	>18-20 hpi	>18-20 hpi	1.29E-08
cell layer	<21-23 hpi	>18-20 hpi	ns	0.045
basal lamina	ns	ns	ns	0.1171
***Ag*^{TEP1KD}**				
blood meal	ns	ns	ns	0.313
cell layer	ns	>24-25 hpi	<21-23 hpi	0.008
basal lamina	ns	ns	ns	0.648
