# Supplementary material for: Kinetics of Plasmodium midgut invasion in Anopheles mosquitoes
Source: PLoS Pathog. 2020 Sep 18;16(9):e1008739. doi: 10.1371/journal.ppat.1008739 (PMC7526910; doi:10.1371/journal.ppat.1008739)
Supplement: S7 Table — (PDF) [file ppat.1008739.s019.pdf]

Table S7. Kruskal-Wallis test of differences in parasite fluorescence intensities between *A. stephensi* (*As*), *A. gambiae* (*Ag*) and *A. gambiae* with silenced *TEP1* (Ag^{TEP1KD}) at all time points.

Ookinete intensity			Kruskal Wallis test	
	As	***Ag***	$\boldsymbol{Ag^{TEP1KD}}$	*P* value
blood meal	> Ag^{TEP1KD},< *Ag*	>Ag^{TEP1KD},> *As*	< *Ag*, *As*	2.00E-17
cell layer	> Ag^{TEP1KD}	> Ag^{TEP1KD}	< *Ag*, *As*	1.00E-15
basal lamina	> Ag^{TEP1KD}	> Ag^{TEP1KD}	< *Ag*, *As*	2.48E-06
