# Supplementary material for: Kinetics of Plasmodium midgut invasion in Anopheles mosquitoes
Source: PLoS Pathog. 2020 Sep 18;16(9):e1008739. doi: 10.1371/journal.ppat.1008739 (PMC7526910; doi:10.1371/journal.ppat.1008739)
Supplement: S8 Table — (PDF) [file ppat.1008739.s020.pdf]

Table S8. Kruskal-Wallis test of differences in parasite fluorescence intensities between *A. stephensi* (*As*), *A. gambiae* (*Ag*) and *A. gambiae* silenced for *TEP1* (*Ag*^{TEP1KD}) at the indicated time points after infection (hpi).

Ookinete fluorescence intensity				Kruskal Wallis test
	As	***Ag***	***Ag***^{TEP1KD}	*P* value
**18-20 hpi**				
blood meal	> *Ag*^{TEP1KD}	ns	*Ag*^{TEP1KD} < *As*	0,00
cell layer	> *Ag*^{TEP1KD}	ns	*Ag*^{TEP1KD} < *As*	6.10E-05
basal lamina	> *Ag*^{TEP1KD}	ns	< *As*	0.03
**21-23 hpi**				
blood meal	> *Ag*^{TEP1KD}	> *Ag*^{TEP1KD}	< *Ag, As*	5.00E-04
cell layer	ns	> *Ag*^{TEP1KD}	< *Ag*	0.02
basal lamina	ns	ns	ns	0.62
**24-25 hpi**				
blood meal	< *Ag*	> *Ag*^{TEP1KD},> *As*	< *Ag*	3.00E-10
cell layer	> *Ag*^{TEP1KD}	> *Ag*^{TEP1KD}	< *Ag*, *As*	6.00E-08
basal lamina	ns	> *Ag*^{TEP1KD}	< *Ag*	2.60E-03
