# Supplementary material for: Kinetics of Plasmodium midgut invasion in Anopheles mosquitoes
Source: PLoS Pathog. 2020 Sep 18;16(9):e1008739. doi: 10.1371/journal.ppat.1008739 (PMC7526910; doi:10.1371/journal.ppat.1008739)
Supplement: S9 Table — (PDF) [file ppat.1008739.s021.pdf]

Table S9. Kruskal-Wallis analyses of parasite fluorescence intensities in *A. stephensi* (*As*), *A. gambiae* (*Ag*) and *A. gambiae* silenced for *TEP1* (Ag^{TEP1KD}), between the different positions blood meal (BM), cellular layer (CL) and basal lamina (BL) at the indicated time points after infection (hpi).

Ookinete fluorescence intensity				Kruskal Wallis test
	**blood meal**	**cell layer**	**basal lamina**	*P* value
As				
18-20 hpi	<BL,CL	>BM	>BM	1.70E-18
21-23 hpi	<CL	>BM	ns	1.21E-07
24-25 hpi	<BL,CL	>BM	>BM	2.30E-09
Ag				
18-20 hpi	<BL,CL	>BM	>BM	4.20E-13
21-23 hpi	<CL	>BM	ns	2.40E-11
24-25 hpi	<BL,CL	>BM	>BM	3.00E-04
Ag^{TEP1KD}				
18-20 hpi	<BL,CL	>BM	>BM	2.50E-07
21-23 hpi	<CL	>BM	ns	1.00E-04
24-25 hpi	<BL,CL	>BM	>BM	7.94E-11
