# Supplementary material for: Kinetics of Plasmodium midgut invasion in Anopheles mosquitoes
Source: PLoS Pathog. 2020 Sep 18;16(9):e1008739. doi: 10.1371/journal.ppat.1008739 (PMC7526910; doi:10.1371/journal.ppat.1008739)
Supplement: S10 Table — (PDF) [file ppat.1008739.s022.pdf]

Table S10. Number of dextran-positive cells in *A. gambiae* and *A. stephensi* mosquitoes at 18-25 h post infection.

	A. stephensi	***A. gambiae***
Records analyzed (n)	11	23
Dextran positive cells (n)	17	52
Dextran positive cells containing a parasite (n)	12	20
Dextran positive cells with an ookinete, %	**71**	**39**
-within the Blood meal, %	**71**	**50**
-within the Cell layer, %	**0**	**31**
