# Supplementary material for: Kinetics of Plasmodium midgut invasion in Anopheles mosquitoes
Source: PLoS Pathog. 2020 Sep 18;16(9):e1008739. doi: 10.1371/journal.ppat.1008739 (PMC7526910; doi:10.1371/journal.ppat.1008739)
Supplement: S11 Table — (PDF) [file ppat.1008739.s023.pdf]

Table S11. Summary of phenotypes *A. stephensi* (*As*), *A. gambiae* (*Ag*) and *A. gambiae* with silenced *TEP1* (*Ag^{TEP1KD}*).

	As	***Ag***	***Ag^{TEP1KD}***
**Infection level**	high	low	high
**Proportion of parasites in Blood meal, %**	~70	~70	~20
**Ookinete speed in Blood meal, um/min**	8.2	3.5	--
**Proportion of parasites in Cell layer, %**	~20	~20	~50
**Ookinete speed in Cell layer, um/min**	0.4	1.8	
**proportion of intercellular ookinetes, %**	~50	~25	~30
**Dextran filled cells contain a parasite, %**	~70	~40	
**Proportion of parasites in Basal lamina, %**	~10	~10	~20
**Ookinete speed in Basal lamina, um/min**	0.3	0.5	--
