# Supplementary material for: Kinetics of Plasmodium midgut invasion in Anopheles mosquitoes
Source: PLoS Pathog. 2020 Sep 18;16(9):e1008739. doi: 10.1371/journal.ppat.1008739 (PMC7526910; doi:10.1371/journal.ppat.1008739)
Supplement: Attachment — Submitted filename: Rebuttal letter.docx [file ppat.1008739.s025.docx]

Reviewer #1.

Q1: The introduction could be improved to provide more background regarding our current understanding of ookinete invasion, harnessing several papers in the early 2000s that were thoroughly summarized by **Baton and Ranford-Cartwright (2005).** Although this reference is cited, I think it is a missed opportunity that wasn’t further discussed to illustrate some of the current gaps in knowledge that are addressed in part through the current manuscript. At one point, it was a more prevalent debate as to whether ookinetes took an intra- or inter-cellular route of invasion.

A: **The text was changed according to suggestion (lines 81-90).**

Q2: Similar to the above comment, I think the discussion should also be expanded to place the results of manuscript in greater context of the previous work. Additional references such as Baton and Ranford-Cartwright (2004), Baton and Ranford-Cartwright (2012), and potentially others would improve the discussion. I also think that these data challenge the “time-bomb” model and should be discussed.

A: **We agree with the reviewer and changed the text accordingly (lines 315-337).**

Q3: I was pleased to see some of the video links in Table S1 and S2. I didn’t watch all of the links, but am concerned by the about of movement during the time course imaging experiments. In my mind this limits the amount of analysis that can be concluded from these imaging experiments, including any kinetics experiments. How were these data examined in the presence of these movements? How can things like ookinete speed then be determined in these experiments? This seems like a pretty big hurdle in the analysis and interpretation of the data.

A: **We apologize that our methodological descriptions were unclear and have better detailed the methods used for determination of parasite position, tracking and movement compensation in the Methods section. We also added a tutorial video to help the access and use of the database (lines 412-416, 456-469).**

Q3: Throughout the manuscript, there are several mentions that TEP1 or mosquito complement impair ookinete invasion. While I do not refute the data provided in several figures of the manuscript, I don’t believe that one can justify that this is entirely direct based on the data provided or other known aspects of previous work from your group, it’s better to leave this open-ended. As a result, I think direct language like “TEP1 inhibits midgut invasion” should be tempered. To me, it seems that these effects are probably a mixture of direct roles of recognition at the basal lamina and indirect roles in the gut given the increased invasion phenotype and TEP1 levels. Loss of TEP1 could significantly influence the composition of the midgut microbiota, where the dysbiosis could potentially help ookinete invasion. I don’t believe that this has been adequately examined. This idea is supported in part as mentioned by Dong et al (2009) for TEP1, and a recent paper by Mitri et al (2020) <https://www.frontiersin.org/articles/10.3389/fmicb.2020.00306/full> where loss of APL1 influenced the abundance of specific bacterial taxa in the gut. This alternative at minimum should be integrated into the revised manuscript.

A: **We thank the reviewer for this comment and changed the text accordingly (lines 163-166, 169-171).**

Q4: The temporal aspects of ookinete invasion success are interesting. I was intrigued by the discussion of the later timepoint (Lines 177-193) and found this very similar to the temporal aspects of TEP recognition discussed in a recent preprint by Kwon et al (<https://www.biorxiv.org/content/10.1101/801480v1>) where they examined the role of the BL as a physical barrier for immune recognition. The degradation patterns and repair of the BL are very similar and should be integrated in the manuscript.

A: **The recent work by Kwon et al. examined the regeneration of the basal lamina which takes place 6-24h after blood meal and coincides with ookinete midgut traversal. This important process may well affect ookinete migration but we did not follow basal lamina in our experiments. Instead of introducing a hand-waving sentence, we believe that the set of tools developed in our work will certainly be valuable for future studies of the link between parasite development and basal lamina regeneration.**

Reviewer #2: Specific, substantial areas to address are the following:

Q1: Lines 188-196 Speculation on Tep1 depletion and two waves. This is to explain two timepoints but there is nothing to back it up, even Tep1 staining, measurement of Tep levels etc

A: **The text was changed according to suggestion (lines 202-213).**

Q2: Line 220 re: differences in speed of motility at lamina and in epithelia in different mosquitoes - why does it point to ‘cellular organisation’ as being the determinant of this. To me this sounds like a difference in cytoskeleton or receptor:ligand combinations is expected. But couldn’t it be some soluble, humeral factor or more general physiological trait? After all, the differences in speed observed in the blood bolus fraction are not expected to be caused by differences in cellular organisation, are they?

A: **The parasite speed is slow when they move through the cellular layer, hence the reference to cellular organization. The molecular basis of this observation (cytoskeletal organization, receptor/ligand combination) will be explored in further studies.**

Q3: Line 229 -232 The parasites with the score between 0 - 0.45 were defined as extracellular, 0.45-0.55 - as intercellular, and higher than 0.55 - as intracellular.
Is this arbitrary - what is the basis for choosing these values? Particularly the intercellular…if, as suggested this is an important category it would be good to have some validation that these are really intercellular

A: **The score that was used to define the position of parasites within the cellular layer was calculated as a normalization of the ratio of the 3D Euclidean distance between the center of a parasite and the nearest nuclei center, and the average distance of nuclei center between them. To validate our scores, we performed visual verification (see the database). Examples of these scores are given in Figure 4 a and b. Furthermore, we provide a 3D projection in XZ and YZ for each record to better visualize the parasite positions. This information was added to the methods section (lines 452-457).**

Q4: Line 293 “while some dextran filled cells contained a parasite, most midgut cells that we observed to host a parasite were dextran-negative, indicating that ookinete invasion damaged and killed only a small proportion of midgut cells” this, to me, seems to be an important finding but I am surprised it is not given more context. This is not my field but I was under the impression that prevailing wisdom was this ‘time bomb’ theory where nearly all cells through which the parasite passes are extruded and apoptose. How do these results weigh up against that? Is there any way to quantify the number of invaded cells relative to the number of cells that get extruded? i.e. is this parasite-induced extrusion occurring in a minority or the majority of cell invasion events? What is a ‘small proportion’?

A: **We thank the reviewer for this suggestion and extended discussion of our results in view of the “time bomb” theory was added, as well as an explanation of the “small proportion” (lines 315-337).**

Q5: Line 310 “ “pioneer” parasites that first reach the basal side of the midgut were rapidly eliminated by the mosquito immune system, and that colonization of the mosquito midgut was initiated at later stages of the infection” again, this would be an important finding but I am not convinced there is enough proof of that in the data reported here in this manuscript.
It seems to me more like a case of back-fitting hypotheses to fit temporal or special observations. That is to say, it is a good place to start investigation of these hypotheses, facilitated by this excellent set of tools, but to accept or reject these would require further experimental corroboration.

A: **We thank the reviewer for this suggestion and changed the text to reflect that more experiments are needed to verify this point (lines 202-213).**

Reviewer #3:

Q1: Lines 138-141: It is unclear how the investigators fitted the cellular layer relative to the nuclei, and how they devised this methodology. This is important because much of the data rely on accurately measuring the location of the midgut lumen, the cell layer, and the basal lamina. More explanation is needed.

A: **We agree with the reviewers and** **added the a more detailed description of thr methods used for the determination of parasite position, tracking and movement compensation in the method section (lines 428-469).**

Q2: Lines 143-160, fig 2e: Notably absent here is any type of statistical analysis that validates the conclusions. Were the trials paired (species and KD treatment) so that comparisons can be made? Moreover, the stated conclusion is “an additional role of TEP1 in inhibition of ookinete midgut invasion.” But the number of parasites in the luminal side of A. gambiae and A. gambiae-TEP1KD mosquitoes does not appear markedly different. Instead, the number of parasites in the cell layer and basal lamina are higher in KD mosquitoes. So, this could be interpreted as TEP1 killing the parasites during cellular traversal and not by preventing invasion. This is where a multivariable statistical analysis would pinpoint where the differences are and would allow for more strongly supported conclusions. Lines 261-263, “we hardly detected any dead parasites in AgTEP1KD mosquitoes, suggesting that TEP1 may be involved in killing parasites within the cellular layer”: the author’s words support the statement I made regarding lines 143-160, fig 2e.

A: **We thank the reviewer for pointing out this important issue. All trials described in our study were unpaired as it is impossible to collect repeated data from the same midgut.** **We added a statistical analysis for the parasite numbers in distinct areas of the cellular level. There is a significant (*p*= 0.0001) increase in cellular parasites in *AgTEP1^KD^*. These results are further corroborated by the apparent lack of dead parasites within the cellular layer. The *p* values and the tests used for statistical analyses were added to Figure 2e.**

Q3: Lines 184-187, fig 3a: The conclusion is that Tep1 eliminates the decrease in the basally located ookinetes and increases the proportion of parasites within the cellular layer. Again, this is unsupported by statistics. The only statistical test that could be used here is that in the basal lamina there is a significant bimodal effect that is not present (statistically speaking) in KD mosquitoes. But the trend is very much the same, and the reason for non-significance in KD mosquitoes appears to be the higher variance, which means the interpretation could be a type 2 error. In that sense, I do not believe the conclusions that ensue the rest of the paragraph are supported.

A: **We agree with the reviewer and modified** **the text to reflect that more experiments are needed to validate this effect of TEP (lines 202-213).**

Q4: Section that begins in line 223, fig 4: It is unclear how the investigators arrived at the math used for figure 4 (and figure 4b is difficult to interpret). This is critical because the math is used to determine parasite location. Perhaps adding the axes (luminal, basal, lateral) to figure 4b plus additional text would make this clearer?

A: **We apologize for this confusion.** **The score was used to define the position of parasites only within the cellular layer. The score is a normalized ratio of the 3D Euclidean distance between the center of a parasite and the nearest nuclei center, and the average distance of nuclei center between them. To validate our scores, we performed visual verification (see the database). Examples for these scores are given in Figure 4 a and b. Furthermore, we provide a 3D projection in XZ and YZ for each record to better visualize the parasite positions. This information was added to the methods section (lines 452-457).**

**Part III – Minor Issues: Editorial and Data Presentation Modifications**

Reviewer #1:

Q1: I am curious with the new microscopy methods and other calculations of ookinete speed provided in the manuscript, can the authors provide any information on how long it takes for ookinetes to traverse the midgut? Even an estimate of minutes vs hours would be informative.

A: **That is a great question. We were eager to estimate this average invasion time but failed to detect any single parasite progressing from the bolus to the basal side of the midgut.** **Our database of images describes parasite dynamics at different time points after infection. Together with parasite position analysis it can provide substantial amount of information related to the invasion process. However, the complexity of the process, manifested in the possibility to traverse the midgut using different routes (through or between the cells), the observation that some parasites remain within cells for hours (at least *ex-vivo*) together with dynamic elimination of parasites at the basal lamina (we do not observe a simple accumulation of parasites at the basal lamina but rather an increase, then decrease and then increase again) did not allow us to calculate the average time necessary for passage from the blood bolus to the basal lamina.**

Q2: For the data presented in Figures 4 and 5, it is not entirely clear what is meant by “extracellular”. Is this considered the midgut lumen? Please clarify.

A: **Parasites located within the cellular layer were characterized by their position relative to the midgut cells. These parasites can be located either between cells, inside cells or just outside the cells (extracellular), but are not far enough from the cellular layer to be considered in the midgut lumen or basal lamina. This is now clarified in the text (lines 241-242) and in the methods section (lines 452-457).**

Q3: Data presented in Figures S8 and S9 is more or less glossed over in the manuscript text. It is curious as to why the intensity of parasite fluorescence is lower in intensity in the TEP1 kd. Could this be an effects of “weaker parasites” surviving when they would ordinarily be killed?

A: **We do not have any experimental data that correlates parasite fluorescence intensity and parasite “health”.**

Q4: Lines 362-363: Why would TEP1 kd increase midgut fragility?

A: **During live imaging of midguts, we noted high midgut fragility in *AgTEP^KD^* mosquitoes. We thought it was important to report it but the molecular mechanisms underlying this phenotype are currently unknown.**

Reviewer #2:

Q1: Line 76 needs citations

A: **The citation was added.**

Q2: Line 88 given that the above statement mentions that APL1,LRIM1 and TEP1 all circulate in hemolymph it is not obvious why crossing 'between' epithelial cells to reach basal lamina would mean they 'thus avoid' TEP1

A: **An earlier report proposed that intracellular nitration of parasites in the midgut cells labels the parasites for binding by TEP1 (Oliveira et al., 2012). Thus, passage between cells will allow the parasites to avoid TEP1 binding.**

Q3: Line 119 state the promoter

A: **The promoter names were added into the text.**

Q4: Line 146 is there a difference in number of ookinetes between Ag and As though? Apparently not, looking at left and mid panels of Fig 2e. So difference between species is noteable at ookinete to oocyst transition? is this discussed.

A: **We agree with the reviewer, we also found no difference in the overall numbers of ookinetes in *Ag* and *As*. Instead, major differences were observed in ookinete dynamics between the two species that may lead to different rates of parasite development and clearance, eventually leading to the observed differences in oocyst loads.**

Q5: Line 152 are these differences significant? P-values and test used?

A: **Yes, statistical significance** ***p* = 0.001, Mann-Whitney t-test. This information was added to the text (lines 160-161 and Figure 2e).**

Q6: There appears to be significantly different expression patterns of GFP (Fig S1) between Ag and As, with the latter being very heterogeneous, including large numbers of cells that do not express the marker? Can this confound/bias the results in terms of cells counted/viewed? – I note this is addressed later, somewhat. But could the presence and absence of expression represent different cell types – if so couldn’t this be problematic in drawing general conclusions across the whole midgut epithelium, for As at least?

A: **Indeed, as previously reported, GFP expression driven by *G12* promoter is highly variable in *As*. However, we were still able to define the midgut cell layer and determine the position of parasites relative to this layer, thus giving us the opportunity to compare between *As* and *Ag*.**

Q7: Line 202 “guid” needs explanation

A: **Guid is a unique identification number given to each record when it is entered into the database. This information was added to the text (line 145).**

Q8: Line 258 states “Differences in distribution were observed for live and dead parasites within the cellular layer” but the legend for Figure S7 states “No correlation was found between the position of the parasite and the level of fluorescence intensity.” When indeed the figure does seem to back up the main text in that it shows an enrichment of black parasite foci that correlate with the midgut epithelial stack. Connecting this point to the point above about surety of calling ‘intercellular’ parasites, I find it curious that there is not difference between the %dead in the intercellular and intracellular classes, indeed they have identical values. Perhaps some of this is confusion is me getting mixed up with ‘position score’ (relating to inter, intra, extra etc.) and height in stack i.e. basal lamina, epithelial or bolus. Either way, if I’m confused so will some readers be, so I would suggest trying to make this a bit clearer.

A: **We thank the reviewer for pointing this out.** **This statement relates to parasites that exhibit fluorescent intensity greater than zero. The parasites that lack fluorescence can only be detected as a dark area within the GFP background of the cellular layer. We changed the text and S7 Fig legend to better explain this point (lines 267-268).**

Q9: Fig 4d is a graph and table of identical I presume. I would leave the graph and label y-axis as “%intercellular parasites”

A: **We thank the reviewer for this suggestion and changed** **Figure 4d accordingly.**

Q9: Line 259 “More dead parasites were found to be located extracellular or intercellular (compare Fig 5c and Fig 4d)” it seems strange to make reader compare values in two different figures to reach a conclusion but if I’ve followed correctly, for all parasites in Ag there is a 27:56.7 ratio of inter:intra whereas for ‘dead only’ it is 1:1 (45.5:45.5). Presumably this is significant?

A: **Fig 4d shows live parasites while Fig 5c describes dead parasites, hence the comparison between the two sets of data. For live parasites in *Ag,* the ration between intercellular to intracellular is indeed roughly 1:2 (27:57), whereas for dead parasites this ratio is 1:1. This difference is significant (*p*=0.009, Mann-Whitney test).**

Q10: I am confused about the sample sizes in Fig 5. In panel b there are 31 mosquitoes looked at. Panel c says 41, which I presume refers to the number of mosquitoes. Were they separate experiments?

A: **We thank the reviewer for this comment.** **In Figure 5b we estimated the proportion of dead parasites from all experiments that contained at least 10 parasites (31 records). In Figure 5c, we analyze the position of dead parasites, so we show analysis of all records that contained dead parasites regardless of total number of the parasites (41 records). We added this information into the Figure legend.**

Q11: Line 287 should this be “one dextran filled cell …with a parasite” or is this one of 20 cells found “in” the midgut had dextran? I must say it’s not clear to me how one efficiently recovers or visualises single cells in a cavity as voluminous as the midgut lumen without a large error.

A: **We apologize for the confusion.** **Since the midgut cells express GFP and their nuclei are stained with DAPI, we can determine the position of the cellular layer and place the dextran filled cells within the cellular layer or outside it. Our results show that out of 53 dextran-positive cells (visualized in several experiments) only one was found in the midgut lumen (Fig 6b). We hope that the introduced text changes help better understanding of our approach (lines 299-301).**

Q12: Line 298 “indicating that parasites refrain from entering an invaded cell.” Are there alternative explanations for this observation other than they are prevented from entering - what if two parasites entering the cell ‘burst’ it, or triggered a very rapid apoptosis that meant they were just not observed?

A: **We agree with the reviewer that although highly unlikely, these alternative explanations cannot be excluded. Therefore, we modified the text accordingly (lines 331-334).**

Reviewer #3:

Q1: Lines 110-112: Was a “new function” of Tep1 really discovered, or did we simply learn more about how Tep1 functions? What is presented seem like a more accurate pinpointing of how Tep1 functions.

A: **We thank the reviewer for this comment and changed the text** **accordingly (lines 119-122).**

Q2: One of the major conclusions of the study is that “the route of ookinete invasion for the same parasite is species-specific and shaped by…”. This could be true, but wouldn’t it be more likely that the difference in invasion efficiency between the two species leads to the phenotypes observed here? Plasmodium berghei does not share an evolutionary history with either A. gambiae or A. stephensi, so it seems unlikely that different invasion strategies evolved.

A: **This is a difficult question as we are indeed studying laboratory models. Therefore, we changed the text according to the reviewer suggestion (lines 117-119).**

Q3: Is it necessary to abbreviate A. gambiae to Ag, A. stephensi to As and P. berghei to Pb in an online only, open access journal? It makes for a clumsy read.

A: **These abbreviations are used both in the text and in the figures. Since we do not use the full names in the figures, we feel that it is easier to follow the abbreviated names.**

Q4. Whenever possible it is best to make definitive statements instead of ambiguous ones. For example, lines 101-103 convey the different rates of development without stating which rate is faster. The answer is known, so why not tell the reader? Another example in the same paragraph: line 108 speaks of “unexpected differences” without providing a clue as to what they are.

A: **We thank the reviewer for this suggestion and** **changed the text accordingly.**

Q5. Line 35: would it be best to use “shaped in part” instead of “shaped”? Undoubtedly there are factors that play a larger role than Tep1.

A: **The text was changed accordingly (lines 31-34).**

Q6. The supplementary figures are presented out of order, making it a more cumbersome read.

A: **Supplementary figures were listed in order of appearance.**
